# Clinical efficacy of Thalidomide combined with PAD Regimen in patients with multiple Myeloma

**DOI:** 10.12669/pjms.39.5.6815

**Published:** 2023

**Authors:** Yu Wang, Qi-hang Man, Chao Li

**Affiliations:** 1 Yu Wang, Department of Hematology, Beijing Aerospace, General Hospital, 100076 Beijing, China; 2 Qi-hang Man, Department of Hematology, Aerospace Center Hospital, 100049 Beijing, China; 3 Chao Li, Department of Hematology, Beijing Aerospace, General Hospital, 100076 Beijing, China

**Keywords:** Thalidomide, PAD regimen, Multiple myeloma, Treatment

## Abstract

**Objective:**

To evaluate the clinical efficacy of thalidomide combined with PAD regimen in patients with multiple myeloma (MM).

**Methods:**

It was a Clinical comparative study. A total of 120 patients with MM were admitted at Beijing Aerospace General Hospital from September 2020 to June 2022 randomly divided into two groups, with 60 patients in each group. The study group was treated with thalidomide combined with a PAD regimen (bortezomib, doxorubicin and dexamethasone), while the control group with a PAD regimen alone. After treatment, the therapeutic effect, adverse drug reactions, bone metabolic markers such as serum alkaline phosphatase (ALP) and osteocalcin (OCN) before and after treatment, as well as T-lymphocyte subsets CD3^+^, CD4^+^, CD8^+^ and CD4^+^/CD8^+^ levels before and after treatment were compared and analyzed between the two groups.

**Results:**

The total efficacy in the study group was 90%, which was significantly higher than 70% in the control group (*p=* 0.00). The incidence of adverse drug reactions was 40% in the study group and 38% in the control group, without statistically significant difference (*p=* 0.85). After treatment, ALP and OCN levels in the study group were significantly higher than those in the control group (ALP, *p=* 0.01; OCN, *p=* 0.00), and CD3^+^, CD4^+^ and CD4^+^/CD8^+^ in the study group also increased significantly compared with those in the control group (CD3^+^, *p=* 0.02; CD4^+^, *p=* 0.00; CD4^+^/CD8^+^, *p=* 0.00).

**Conclusion:**

Thalidomide combined with a PAD regimen is definitely effective in patients with MM, it can obviously improve immune function and bone salt metabolism, with no increase in adverse reactions but high safety and effectiveness.

## INTRODUCTION

Multiple myeloma (MM) is a common hematological disease, accounting for 10% of all tumors of the hematopoietic system.^1^ This disease is also a malignant clonal plasma cell disease. Most of such patients will develop bone diseases, manifested as bone pain, pathological fractures and nerve compression syndrome, which seriously affect their quality of life.^2^ At present, chemotherapy is often used to treat MM. Although chemotherapy can alleviate the condition, the remission rate is not high. With the introduction of novel drugs, the therapeutic effect in patients with myeloma has been significantly improved. However, this disease is considered incurable and shows considerable heterogeneity in clinical manifestations, course of disease and survival rate.^3^

PAD regimen consists of bortezomib, doxorubicin and dexamethasone. Bortezomib has a strong killing effect on myeloma cells, and is widely used in the adjuvant therapy of chemotherapy for MM.^4^ Nevertheless, for the treatment of MM, combined therapy usually has a deeper response and better long-term outcomes than chemotherapy alone.^5^ Thalidomide is a glutamate derivative, which not only has immunomodulatory and anti-inflammatory effects, but also can inhibit angiogenesis and reduce microvessel density in tumors, so as to play an anti-tumor role.^6^ In our study, thalidomide combined with a PAD regimen was used to treat patients with MM, which achieved certain clinical results.

## METHODS

It was a Clinical comparative study. A total of 120 patients with multiple myeloma (MM) were admitted at Beijing Aerospace General Hospital from September 2020 to June 2022 randomly divided into two groups, with 60 patients in each group. In the study group, there were 32 males and 28 females, aging 55-70 years (average, 60.75 ± 6.74 years). The control group included 35 males and 25 females, with an age of 58-69 years (average, 62.70 ± 6.61 years). The general data of the two groups showed no significant differences, suggesting comparability (Table-I).

**Table-I:** Comparison of general data between study group and control group (*χ̅*±*S*) n = 60.

Index	Study group	Control group	t/χ^2^	*p*
Age (year)	60.75 ± 10.74	62.70 ± 10.61	0.97	0.32
Male (%)	32 (53.3%)	35 (58.3%)	0.30	0.58
DS stage				
Stage-I	16 (26.7%)	14 (23.4%)	0.18	0.67
Stage-II	19 (31.7%)	23 (38.3%)	0.59	0.44
Stage-III	25 (41.6%)	23 (38.3%)	0.14	0.71
***Osteolytic lesion***				
2	16 (26.7%)	19 (31.7%)	0.36	0.55
3	24 (40%)	23 (38.3%)	0.04	0.85
> 3	20 (33.3%)	18 (30%)	0.15	0.70

p> 0.05.

### Ethical Approval

The study was approved by the Institutional Ethics Committee of Beijing Aerospace General Hospital (No.: AF/SQ-02/01.0; Date: April 15, 2020), and written informed consent was obtained from all participants.

### Inclusion criteria

- Age< 70 years;
- Meeting the diagnostic criteria of MM^7^;
- At least two or more osteolytic lesions by bone scanning;
- Durie-Salmon (DS) stage I-III^8^;
- Good physical condition with the ability to take care of themselves (KPS score ≥ 70);
- Complete case data and follow-up data.

### Exclusion criteria:

- Combined with other hematological diseases;
- Combined with malignant tumors of other organs;
- Poor physique and intolerance to treatment;
- Mental disorders and nervous system abnormalities, or inability to cooperate with the study for other reasons;
- Hepatic and renal dysfunction;
- Allergy or intolerance to drugs used in this study;
- Recent oral administration of drugs that affect the results, such as hormones and immunosuppressants; pregnancy or lactation.

### Treatment methods

Blood cell analysis and detection of hepatic and renal function were performed in both groups, and the abnormal indexes were corrected correspondingly. One day before chemotherapy, hydration was conducted. During chemotherapy, the detection was carried out, and the patients were treated with antiemetic therapy, hepatic and renal function-protecting therapy and fluid replacement.

The study group was treated with thalidomide combined with a PAD regimen (bortezomib, doxorubicin and dexamethasone): bortezomib (1.3 mg/m^2^/d) was injected intravenously on the 1st, 4th, 8th and 11th day; dexamethasone (20 mg) was injected intravenously daily on the 1st~4th and 8th~11th day; doxorubicin (15 mg/m^2^/d) was injected intravenously on the 1st~4th day. Additionally, thalidomide was given at the initial stage of inductive chemotherapy, 25 mg orally, three times/d. Treatment was continued for 2-8 courses (every 11 day), with an interval of three weeks.

The control group was treated with a PAD regimen alone (bortezomib, doxorubicin and dexamethasone). After treatment, both groups underwent a routine blood test, detection of hepatic and renal function, electrocardiography (ECG) and chest X-ray, during which antibiotics were given according to the specific condition of the patients.

### Observation indexes:

### Evaluation of clinical efficacy

After treatment, the clinical efficacy of the two groups was evaluated as: complete remission (CR): after treatment, serum M protein test was negative, which lasted for six weeks, plasma cell tumors disappeared, the content of bone marrow plasma cells < 5%, and osteolytic lesions were not aggravated; nearly complete remission (nCR): after treatment, immunofixation electrophoresis was positive, plasma cell tumors disappeared, the content of bone marrow plasma cells < 5% and osteolytic lesions were not aggravated; partial remission (PR): after treatment, the content of serum M protein decreased by more than 50%, which lasted for > six weeks, 24-hour urinary light chain < 200 g or its decrease > 90% for more than six weeks, the reduction of plasma cell tumor > 50%, and osteolytic lesions were not aggravated; mild response (MR): after treatment, the content of serum M protein decreased by 25%~49%, which lasted for > six weeks, and 24-h urinary light chain decreased by 50%~89%; no change (NC): after treatment, the clinical symptoms and signs ranged from mild remission to progression; disease progression (DP): after treatment, absolute serum M protein content increased by more than 5 g/L, absolute 24-h urinary light chain increased by 200 mg, absolute bone marrow plasma cell content increased by 10%, and osteolytic lesions were aggravated. Total effective rate = (CR + nCR + PR)/total cases × 100%.

### Evaluation of adverse drug reactions

The adverse drug reactions of the two groups within one month after treatment were recorded, including bone marrow suppression, hair loss, gastrointestinal reactions, hepatic and renal dysfunction and fever.

### Comparison of bone metabolic markers before and after chemotherapy

About 5 mL of venous blood was collected before and after chemotherapy, followed by centrifugation and serum isolation. The levels of serum alkaline phosphatase (ALP) and osteocalcin (OCN) were detected using an enzyme-linked immunosorbent assay (ELISA).^4^ Analysis of immune state: Fasting blood was collected at the morning before and after treatment to detect the levels of T-lymphocyte subsets CD3^+^, CD4^+^, CD8^+^ and CD4^+^/CD8^+^ determined by flow cytometry, and the differences between the two groups before and after treatment were compared and analyzed.

### Statistical analysis

All data were statistically analyzed using SPSS 20.0. The measurement data were expressed as (*χ̅*±*S*). Two-group independent samples t-test were used for inter-group comparison, paired t-test for intra-group comparison, and χ^2^ test for rate comparison. P< 0.05 was considered as statistically significant.

## RESULTS

The comparison of clinical efficacy between the two groups is shown in Table-II. It was suggested that the total efficacy in the study group after treatment was 90%, which was significantly higher than 70% in the control group (*p=* 0.00).

**Table-II:** Comparison of clinical efficacy between the two groups (*χ̅*±*S*) n = 60.

Group	CR	nCR	PR	MR	NC	PD	Total effective rate*
Study group	27	15	12	6	0	0	54 (90%)
Control group	21	13	8	11	4	3	42 (70%)
χ^2^							7.50
p							0.00

* p< 0.05.

The comparison in the incidence of adverse drug reactions after treatment between the two groups revealed that the incidence of adverse drug reactions was 40% in the study group and 38% in the control group, without statistically significant difference (*p=* 0.85) (Table-III).

**Table-III:** Comparison of adverse drug reactions between the two groups after treatment (*χ̅*±*S*) n = 60.

Group	Hair loss	Hepatic and renal dysfunction	Fever	WBC reduction	Gastrointestinal reactions	Incidence*
Study group	7	5	2	6	4	24 (40%)
Control group	5	4	1	7	6	23 (38%)
χ^2^						004
*p*						0.85

* p> 0.05.

Before treatment, ALP and OCN levels presented no significant differences between the two groups (*p>* 0.05). After treatment, ALP and OCN levels in the study group were significantly higher than those in the control group (ALP, *p=* 0.01; OCN, *p=* 0.00), as seen in (Table-IV).

**Table-IV:** Comparison of bone metabolic markers before and after treatment *χ̅*±*S*) n = 60.

Index	Time	Study group	Control group	t	p
ALP (U/L)	Before treatment	86.31 ± 13.09	86.79 ± 12.41	0.21	0.84
	After treatment*	115.64 ± 21.39	105.28 ± 22.27	2.59	0.01
OCN (ug/L)	Before treatment	15.93 ± 4.68	15.65 ± 4.37	0.34	0.73
	After treatment*	24.57 ± 6.82	20.74 ± 5.13	3.29	0.00

* p< 0.05.

Before treatment, the two groups showed no significant differences in CD3^+^, CD4^+^, CD8^+^ or CD4^+^/CD8^+^ level (*p>* 0.05). After treatment, CD3^+^, CD4^+^ and CD4^+^/CD8^+^ in the study group increased significantly compared with those in the control group (CD3^+^, *p=* 0.02; CD4^+^, *p=* 0.00; CD4^+^/CD8^+^, *p=* 0.00). However, no obvious change was detected in CD8+ (*p=* 0.79) (Table-V).

**Table-V:** Comparison of T-lymphocyte subset levels before and after treatment *χ̅*±*S*) n= 60.

Index	Time	Study group	Control group	t	p
CD3+ (%)	Before treatment	40.82 ± 5.83	40.76 ± 6.05	0.06	0.95
	After treatment*	46.29 ± 5.70	43.91 ± 5.53	2.32	0.02
CD4+ (%)	Before treatment	24.96 ± 4.81	24.85 ± 4.48	0.13	0.89
	After treatment*	33.42 ± 5.33	27.69 ± 5.17	5.98	0.00
CD8+ (%)	Before treatment	23.54 ± 3.08	23.61 ± 3.15	0.12	0.78
	After treatment	23.46 ± 3.27	23.38 ± 3.12	0.14	0.79
CD4+/CD8+	Before treatment	1.32 ± 0.24	1.40 ± 0.31	1.58	0.12
	After treatment*	1.79 ± 0.18	1.57 ± 0.14	7.47	0.00

* p< 0.05.

## DISCUSSION

Our study showed that the total effective rate of thalidomide combined with PAD regimen in patients with MM was 90%, which was significantly higher than 70% in the control group (*p=* 0.00). The incidence of adverse drug reactions was 40% in the study group and 38% in the control group, without statistically significant difference (*p=* 0.85). MM has extensive bone destruction and abnormal bone metabolism. Bone destruction is mainly caused by the imbalance between osteolysis and osteogenesis.

MM is characterized by extensive osteolytic destruction, abnormal rise in monoclonal plasma cell ratio, osteolytic lesions, pathological fractures, anemia, hypercalcemia, nervous system damage, etc.^9^ In addition, monoclonal M protein increases in the patients, which inhibits the synthesis of polyclonal immunoglobulin and increases the risk of infections. It is incurable in the current clinical treatment. Although significant progress has been made in the treatment in the past few decades, satisfactory results still cannot be achieved^10^, seriously threatening the health of patients. In the last two decades, with the emergence of novel drugs, the five years survival rate of MM can only be close to 50%.^11^ At present, chemotherapy is still the main treatmentli for MM^12^, and the commonly used regimens include VAD, PAD, MP, etc.

It has been confirmed that the effect of the PAD regimen on MM is significantly better than that of the VAD regimen.^13^ Bortezomib is a protease inhibitor, which can bind to threonine at the active site of proteasome and inhibit proteasome activity, thus suppressing the degradation of nuclear factor-κB (NF-κB) and inhibitory factor-κB (I-κB) and inducing the apoptosis of tumor cells.^14^ Sonneveld et al.^15^ have pointed out in their study that bortezomib can coordinate the interaction between bone marrow tumor cells and stromal cells, so drug resistance hardly occurs and patients with multiple adverse cytogenetic abnormalities cannot benefit from these drugs. Glucocorticoids are the basis of the treatment of MM. Their compatibility with novel drugs improves the clinical response rate.^16^ Another study^17^ has found that bortezomib has a synergistic effect with dexamethasone, and can reverse the resistance of MM to melphalan, doxorubicin, etc.

The incurability of MM urges people to actively explore more favorable treatment regimens. According to the meta-analysis of Aguiar et al., the multi-target regimen has more obvious advantages than the chemotherapy regimen alone.^18^ Immunotherapy that improves the immune state of the body is changing the treatment for patients with MM at all stages^19^, and achieves obvious effects. Thalidomide is a glutamate derivative, whose mechanisms include sedation and analgesia; immunomodulatory and anti-inflammatory effects and inhibition of angiogenesis and anti-tumor effect. Some cytokines, such as vascular endothelial growth factor and fibroblast growth factor, are stimulants of angiogenesis.

They bind to specific receptors to stimulate signal transduction and cause the proliferation of endothelial cells. This product can reduce their secretion and thereby inhibiting blood vessels. The metastasis and malignant transformation of tumor cells are related to the adhesion and angiogenesis of tumor cells and vascular endothelial cells. Thalidomide cannot only inhibit angiogenesis, but also reduce the synthesis of integrin subunits, which is also one of its anti-tumor mechanisms. Moreover, it reduces the microvessel density in tumors through the COX-2 pathway rather than inhibiting angiogenesis, so as to inhibit the proliferation of tumor cells.^20^

The phase three clinical trial of Tacchetti et al. confirmed that the combined regimen of bortezomib and thalidomide was superior to the chemotherapy regimen alone.^21^ Stork et al.^22^ believe that bortezomib combined with thalidomide has an obvious short-term effect, and the long-term follow-up has verified that the combination with thalidomide provides additional advantages for the OS of patients. Jung’s study^23^ has confirmed that thalidomide + dexamethasone-based regimen has great advantages in patients with recurrent/refractory MM (RRMM). Li et al.^24^ believe that there is no increase in adverse reactions after thalidomide combined with chemotherapy, but thrombosis is still an important consideration in the treatment of MM. Furthermore, appropriate risk stratification and vigilant thrombosis prevention are essential to prevent this complication.

The combined effect of osteogenic differentiation disorders and osteoclastic hyper function leads to bone destruction. ALP and OCN mainly reflect the activity of osteoblasts.^25^ It has been pointed out that the imbalance of T-lymphocyte subsets is closely related to the occurrence and development of MM. The main manifestations of the patients include the decrease in CD4^+^ level and the increase in CD8^+^ level.^26^

### Limitations

It includes asmall sample size and short follow-up time. In the future study, the number of samples will be gradually increased, the follow-up time will be extended, and the advantages and long-term effects of this treatment regimen will be further objectively evaluated.

## CONCLUSION

Thalidomide combined with a PAD regimen is effective in patients with multiple myeloma, and it can obviously improve immune function and bone salt metabolism, with no increase in adverse reactions but high safety and effectiveness.

### Authors’ Contributions:

**YW:** Designed this study and prepared this manuscript, and are responsible and accountable for the accuracy and integrity of the work.

**QHM:** Collected and analyzed clinical data.

**CL:** Data analysis, significantly revised this manuscript.
